# Deconstructing Pilot Contamination Attacks: A Two-Stage Threat Model for MIMO Systems

**DOI:** 10.3390/s26154935

**Published:** 2026-08-04

**Authors:** Abdallah Farraj

**Affiliations:** Department of Electrical Engineering, Texas A&M University-Texarkana, RELLIS Campus, Bryan, TX 77807, USA; afarraj@tamut.edu

**Keywords:** physical-layer security, MIMO, pilot contamination attack, channel estimation, parameterized threat modeling, adversarial wireless communications, precoding poisoning, eavesdropping

## Abstract

Pilot contamination attacks (PCAs) pose a great physical-layer threat to modern multiple-input–multiple-output (MIMO) systems by exploiting channel reciprocity to corrupt channel state information estimation. While existing literature acknowledges this vulnerability, precise parametric threat models that capture the transition from active channel estimation poisoning to data exploitation remain scarce. This article addresses this gap by developing a novel, physical-layer parameterized PCA framework structured as a two-stage operational attack: channel estimation poisoning and adaptive information contamination. We formulate an algorithmic attack strategy that systematically manipulates base transceiver station precoding and beamforming weights. This formulation allows us to quantify the precise degradation of the system through the lens of the confidentiality, integrity, and availability (CIA) triad, specifically mapping the adversary’s security gains against legitimate users’ signal-to-noise ratio degradation. Finally, we leverage this parametric threat model to outline a qualitative roadmap of actionable detection vectors and structural mitigation strategies. The proposed algorithmic attack serves as an evaluation benchmark and an analytical baseline for conceptualizing resilient architectures in emerging physical-layer security frameworks.

## 1. Introduction

The widespread expansion of wireless communication systems has transformed modern society, but this rapid growth also brings significant vulnerability to cyber attacks. Because the wireless medium is open by nature, it is highly susceptible to malicious interference. For operators and researchers, it is very important to understand the impact of these attacks in detail; without this comprehensive understanding, we cannot design the necessary safeguards to protect our wireless networks. As we move toward more advanced communication systems, the necessity to secure these systems against sophisticated threats becomes a primary requirement for infrastructure sustainability [1,2].

In the study of information security, we usually refer to the confidentiality, integrity, and availability (CIA) triad. When we apply this model to wireless communication systems, the technical challenges are unique; for example, confidentiality ensures that the data sent over the air is not intercepted by an unauthorized listener, integrity guarantees that the transmitted signal is not modified or corrupted by a malicious actor during propagation, and availability ensures that the network remains accessible to legitimate users, even when there is intentional interference or jamming.

While most security protocols are implemented at higher layers of the network stack, attacks targeting the physical layer are particularly dangerous [3,4,5,6]. These attacks exploit the fundamental radio frequency characteristics of the wireless channel. For example, jamming can completely block the communication by increasing the noise floor, while spoofing allows an attacker to pretend to be a legitimate node. Because these threats happen at the hardware and waveform levels, they can bypass traditional software-based encryption. This makes the physical layer a critical point of failure in modern wireless systems [7].

In modern multi-antenna wireless communication, such as multiple-input–multiple-output (MIMO) topologies, the base transceiver station (BTS) requires accurate channel state information (CSI) to perform effective beamforming and precoding. This CSI is typically obtained during a pilot training phase, where a legitimate node sends a known pilot sequence to help the BTS estimate the channel and focus the signal toward that user.

A very specific and dangerous threat in these environments is the pilot contamination attack (PCA) [8,9]. This attack occurs when an active adversary transmits the identical pilot sequence synchronously with the legitimate user [10]. This simultaneous transmission causes the BTS to get a poisoned and incorrect estimation of the channel. As a result, the precoding calculations and the beamforming process become corrupted; thus, the BTS might inadvertently direct private data toward the attacker instead of the legitimate user, or the downlink signal quality for the legitimate user might drop significantly due to this attack [11,12].

Therefore, it is essential to deeply understand how the PCA works in order to develop better detection and mitigation strategies. Although other works have discussed this topic, there are still gaps in the current literature, especially regarding how these attacks leverage physical-layer properties of the communication environment in order to conduct successful attacks and when defending against intelligent, adaptive adversaries [13].

This article aims to analyze these mechanisms and provide insights into the vulnerabilities of current channel estimation methods, which is a necessary step to build more resilient and secure wireless architectures. Specifically, we develop a physical-layer-based approach to conduct a poisoning attack on channel estimation in MIMO systems; then, the cybersecurity implications of the PCA are discussed and quantified. An algorithmic attack formulation is also presented, and mitigation strategies to counter this type of attack are discussed.

To focus our analysis on the fundamental vulnerabilities of the underlying signaling protocols, this study investigates a localized, baseline network deployment. By evaluating a baseline configuration without initial propagation artifacts like large-scale path loss, user mobility profiles, or slot synchronization errors, we mathematically isolate the raw interaction between legitimate channel estimation and active adversarial positioning. This targeted configuration provides a rigorous mathematical baseline required to characterize the performance boundaries of the attack before scaling to complex network deployments.

While contemporary studies focus on the macroscopic impacts of pilot spoofing, they frequently treat the adversary as a static noise source or rely on perfect prior channel knowledge. They lack a parameterized, two-stage operational framework that maps physical channel dynamics directly to algorithmic vulnerabilities across the entire CIA triad. This article bridges this critical gap. Unlike classical frameworks that treat injection as static noise, our approach establishes a tunable attack vector via an explicit weight configuration. This allows the adversary to dynamically focus spatial power limits between intercept maximization and link disruption.

The contributions of this article are summarized as follows:

- We develop a parametrized PCA based on physical-layer security principles, presenting a unique approach to attack development.
- We divide the development of the attack into two distinct operational stages: the channel estimation poisoning stage and the contamination attack stage.
- We quantify and analyze the specific impact of this attack on the core pillars of security: confidentiality, integrity, and availability.
- We provide an algorithmic attack formulation that serves as a theoretical framework for attack development, which consequently aids in conceptualizing detection and mitigation mechanisms.

Unlike standard pilot spoofing paradigms that treat the injection of malicious pilots as a standalone interference event, the novelty of this work lies in mathematically connecting the optimization of the attack weight to quantifiable cybersecurity outcomes. The standard literature largely focuses on spoofing mechanics and detection. In contrast, our two-stage framework bridges the gap by mathematically proving how an actively optimized attack vector in the channel estimation poisoning phase dictates the exact severity of the physical-layer security degradation in the pilot contamination phase, specifically through targeted signal-to-noise ratio (SNR) reduction, bit error rate (BER) inflation, and induced outage probabilities at the legitimate receiver. No prior work provides this end-to-end mathematical mapping from deterministic pilot optimization to closed-form cybersecurity degradation.

## 2. Background

Modern wireless networks rely heavily on accurate CSI to achieve high data rates and reliable performance. In standard time-division duplex (TDD) MIMO systems, this CSI is acquired through a pilot-based uplink training phase. During this step, each legitimate user transmits a predefined, orthogonal pilot sequence, and the BTS observes these incoming signals to estimate the individual channel characteristics for each user in its service area.

### 2.1. The Operational Mechanism of Pilot Contamination

A PCA directly exploits the pilot-based training process. Instead of acting as a conventional jammer that floods the channel with random noise, an active adversary acts as a smart spoofer. The attacker transmits a signal that is perfectly aligned with the legitimate user’s pilot sequence. Because these signals are sent simultaneously during the training phase, the BTS cannot separate them. Instead, the BTS observes a superposition of both the legitimate and the malicious users’ signals [14]. During the uplink training phase, the simultaneous transmission of identical pilots confuses the BTS [15]. Conceptually, the resulting poisoned channel estimate for a target user *k* can be expressed as

(1) $${\hat{h}}_{k}=h_{k}+\rho h_{a}+\tilde{n}$$

where $h_{k}$ represents the true channel vector of the legitimate victim, $h_{a}$ is the channel vector of the attacker, ρ denotes the relative scaling factor of the attack power, and $\tilde{n}$ represents the relevant additive white Gaussian noise (AWGN) component at the BTS.

When the system moves to the downlink data transmission phase, the BTS uses this corrupted estimate ${\hat{h}}_{k}$ to calculate its beamforming vectors and power allocation. Because the estimate contains the attacker’s channel signature ($h_{a}$), the resulting signal beam is distorted and part of the beamforming gain is directed toward the attacker’s location instead of focusing solely on the legitimate user. This creates performance degradation, inter-user interference, and a risk of information leakage.

MIMO deployments are especially vulnerable to this threat due to structural constraints in practical networks [16]. Ideally, every user in a wireless network would use a completely unique pilot sequence. However, because the coherence interval of the wireless channel is limited, the total number of available orthogonal pilots is also limited. This shortage forces neighboring cells to reuse the same set of pilot sequences.

Adversaries can exploit this pilot reuse vulnerability. For example, an attacker located near cell edges can target shared pilots across adjacent cells, allowing them to degrade the performance of multiple BTS at the same time. Furthermore, unlike conventional noise jamming, which can be mitigated by adding more antennas at the BTS to average out the noise, PCA scales directly with the network infrastructure. Because the BTS uses its antenna array to actively focus power toward the contaminated channel estimate, the effectiveness of the attack can remain strong even as the number of BTS antennas grows. This makes PCA a critical threat for next-generation MIMO deployments.

Further, more sophisticated adversaries can deploy multi-stage operational strategies that combine passive learning with active contamination. For example, an attacker may first passively monitor the network across several coherence intervals. By doing this, the attacker can infer specific pilot patterns, user scheduling intervals, and power levels. Once done, the attacker transmits timed pilot replicas that maximize impact while blending into the background to avoid traditional detection mechanisms.

### 2.2. Literature Survey and Gap Analysis

The vulnerability of multi-antenna systems to physical-layer exploitation is a well-documented concern in contemporary research. In the foundational literature, Refs. [11,12] analyze the fundamental susceptibility of MIMO configurations to PCAs, where secrecy capacity bounds are explored. Also, Ref. [17] evaluates the macroscopic impacts of PCAs within multi-user networks, deriving achievable performance bounds under mutual interference. To contextually evaluate the baseline data degradation downstream from these attacks, foundational models often examine receiver performance under non-idealities; for example, classical performance analyses establish the mathematical foundations for bit error rate behaviors over linear detectors subject to imperfect channel estimation errors and residual hardware noise tracking. While these analytical guidelines map general vulnerability limits under passive estimation errors, they assume static noise parameters and do not account for an active, intelligent adversary targeting the processing architecture.

On the defensive front, researchers focus on decoupling legitimate and adversarial channel components. For example, Ref. [18] proposes a data-aided secure transmission scheme that leverages data symbols alongside traditional pilots to mitigate the impact of the attack, focusing on neutralizing the attacker’s beamforming gains during the downlink phase. More recently, Ref. [19] provides a comprehensive survey detailing physical-layer authentication methods designed to detect contamination attempts during the uplink training phase. However, these detection and mitigation frameworks often rely on the assumption of static attacker behaviors or require perfect statistical channel knowledge prior to the attack.

Despite these insights, a critical gap persists in the existing literature. Contemporary studies frequently treat the PCA as a localized, single-step event or evaluate its consequences solely through macroscopic metrics like secrecy rate degradation. They lack a two-stage operational framework that maps dynamic physical channel attributes directly to algorithmic vulnerabilities across the entire CIA triad. Specifically, the precise progression from initial channel estimation poisoning to data exploitation remains unaddressed. This article bridges this gap by developing a parameterized, physical-layer threat model structured as a two-stage operational attack. By mapping the direct relationship between the malicious user’s gains and the legitimate victim’s degradation, our framework provides a parametric benchmark required to synthesize resilient, adaptive physical-layer defenses.

While MIMO linear detectors are sensitive to imperfect CSI, the standard literature primarily treats these imperfections as benign phenomena arising from estimation errors or hardware impairments [20,21,22]. These studies typically evaluate system reliability under the assumption that channel estimation errors are stochastic and unavoidable consequences of noisy environments. However, our threat model fundamentally diverges from these frameworks: rather than treating imperfect CSI as a stochastic estimation error, our work models CSI corruption as a deterministic, mathematically optimized PCA designed by an active adversary to structurally degrade the performance metrics of the legitimate user.

## 3. Channel Estimation Poisoning

To mathematically isolate the raw interaction between legitimate channel estimation and active adversarial implications of a PCA, this analysis employs a baseline configuration without initial propagation artifacts like large-scale path loss, user mobility profiles, or slot synchronization errors. By assuming a single-cell, flat-fading MIMO environment, we establish the fundamental theoretical limits of the attack vector.

By leveraging the physical symmetry of the radio channel, transceiver stations can achieve high-resolution downlink beamforming without the excessive overhead of CSI feedback. In TDD systems, the uplink and downlink share the same frequency spectrum and are separated only by time. Thus, the electromagnetic wave propagation path is similar in both directions within the coherence time of the channel. Therefore, the BTS can directly use the channel estimates obtained from uplink pilot training to design its downlink precoding matrices.

### 3.1. System Model

**Figure 1:**
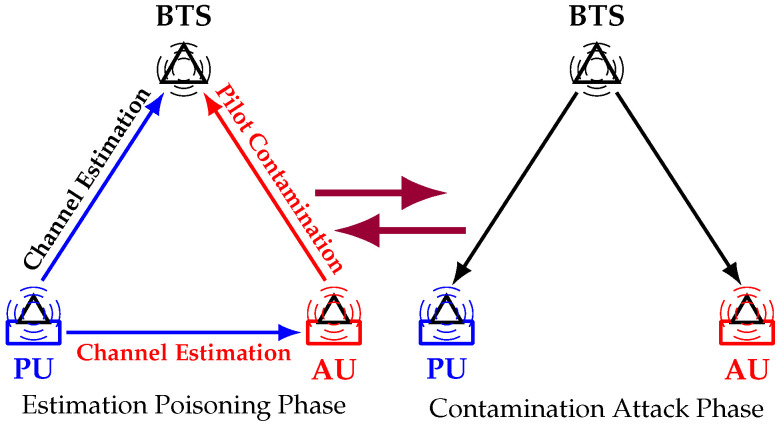
Pilot contamination attack setup.

Consider a MIMO wireless system with a legitimate unit, called the primary user (PU); an adversarial user (AU); and a BTS. The AU’s objective is to conduct a PCA on the PU’s signal in order to compromise the downlink transmission from the BTS to the PU. As illustrated in Figure 1, the PCA is conducted in two stages: channel estimation poisoning and pilot contamination. In the channel poisoning stage, the AU crafts an attack vector to mislead the BTS about the channel gain between the PU and the BTS; however, during the contamination stage, the AU reaps the benefits of the attack through improved signal quantity at its end and/or degraded signal at the PU.

**Figure 2:**
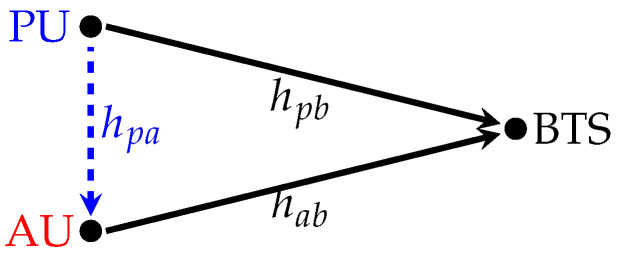
Environment model.

Consider a BTS with *M* antennas. Let $h_{pb}\in C^{M\times 1}$ represent the uplink channel vector from a single-antenna PU to the *M*-antenna BTS, and let $h_{ab}\in C^{M\times 1}$ be the channel of the AU with the BTS as shown in Figure 2. Under the assumption of reciprocity, the downlink channel $h_{bp}$ is simply the transpose of the uplink channel; i.e., $h_{bp}=h_{pb}^{T}$. Similarly, the channel between the PU and the AU is denoted $h_{pa}\in C^{1\times 1}$. Also, let ℜ{z} and ℑ{z} denote the real and imaginary parts of $z\in C^{1\times 1}$, respectively. We adopt a block-fading model where the channel vector remains constant over a specific coherence interval. In the context of this work, it is assumed that both the uplink pilot burst and the subsequent downlink data burst are transmitted within the same coherence interval.

To establish an analytical baseline for this threat model, the active adversary is modeled under a worst-case security boundary scenario. The AU is assumed to maintain slot-level time and frequency synchronization with the legitimate transmissions and holds a local tracking estimate of its own channel vector $h_{ab}$ relative to the BTS. Furthermore, the AU is subject to a peak power constraint of $P_{\max}$. To execute its optimization loop without prior oracle-level knowledge, the AU is assumed to infer the PU’s channel direction $h_{pb}$ over time by passively eavesdropping on open-loop pilot transmissions and reference preambles across consecutive channel coherence intervals. This operational baseline ensures that the derived security limits reflect the absolute performance boundaries of the underlying signaling protocol.

### 3.2. Downlink Precoding and Indirect Channel Estimation

The precoding process begins with the PU transmitting a known pilot signal, $s_{p}$, over the channel. The received signal at the BTS, $y_{b}$, is given by

(2) $$y_{b}=\sqrt{P_{p}}h_{pb}s_{p}+n_{b}$$

where $P_{p}$ is the transmit power of the PU, $|s_{p}{|}^{2}=1$, and $n_{b}∼\mathrm{CN}\left(0,\sigma^{2}I_{M}\right)$ represents the AWGN at the BTS. The BTS applies an estimation algorithm (e.g., least squares or minimum mean square error) to derive the channel estimate ${\hat{h}}_{pb}$. Once ${\hat{h}}_{pb}$ is obtained, the BTS utilizes this estimate to calculate the precoding weight vector $w_{b}\in C^{1\times M}$. The goal is to construct a beam that maximizes the SNR at the PU. In a typical TDD system, the BTS employs channel inversion or zero-forcing precoding to facilitate reception at the PU. For maximum ratio transmission (MRT), the BTS sets the weights to the conjugate of the estimated channel as [23]

(3) $$w_{b}=\frac{{\hat{h}}_{pb}^{H}}{∥{\hat{h}}_{pb}∥}.$$

This precoding vector ensures that the signals from all *M* antennas arrive at the PU in-phase, providing a beamforming gain of *M*.

We now investigate how an AU can infer the CSI, especially the channel gain, between a PU and a BTS [24,25]. We assume channel reciprocity and a quasi-static fading environment where the channel coefficients remain constant over the duration of a frame. The estimation process executed by the AU occurs in two distinct steps, leveraging the legitimate protocol exchange between the PU and the BTS. Upon estimating ${\hat{h}}_{pb}$, the BTS transmits a known signal $s_{b}$ to the PU. The transmitted beamforming signal vector at the BTS is formulated as $\sqrt{P_{b}}w_{b}^{H}s_{b}$, where $P_{b}$ is the transmit power of the BTS. The adversary intercepts this broadcast, with its received scalar signal, $y_{a}=\sqrt{P_{b}}w_{b}h_{ab}s_{b}+n_{a}$, where $n_{a}∼\mathrm{CN}\left(0,\sigma^{2}\right)$ is the noise signal present at the AU. Simplifying the expression, $y_{a}$ is represented as

(4) $$y_{a}=\sqrt{P_{b}}\frac{{\hat{h}}_{pb}^{H}}{∥{\hat{h}}_{pb}∥}h_{ab}s_{b}+n_{a}.$$

The AU can independently estimate the link between the PU and the BTS using the BTS’s preamble signals. Given that $s_{b}$ is a known sequence (e.g., a synchronization signal), the AU isolates the target projection parameter using

(5) $$\frac{{\hat{h}}_{pb}^{H}h_{ab}}{∥{\hat{h}}_{pb}∥}\approx \frac{y_{a}}{\sqrt{P_{b}}s_{b}}$$

under high SNR conditions ($\sigma^{2}\to 0$). The AU keeps repeating this process to generate more accurate estimates of the channel configuration boundaries. As shown, the BTS reliance on open-loop precoding based on uplink pilots can create a potential vulnerability where the AU effectively uses the precoding information from the BTS to gain an estimation baseline.

### 3.3. Poisoning Attack

Given its estimate baseline as demonstrated in (5) and the PU’s known pilot signal $s_{p}$, the AU can design a channel estimation poisoning attack. During the PU’s training phase, the AU synchronously transmits the same pilot sequence $s_{p}$ along with the PU’s pilot transmission. However, instead of only sending $s_{p}$ over the channel, the AU transmits $w_{a}s_{p}$, where $w_{a}\in C^{1\times 1}$ and $|w_{a}|=1$ is the parameterized attack weight vector. $w_{a}$ needs to be determined with the objective of conducting the pilot contamination attack as detailed in Section 4.

With the PU sending $s_{p}$ and the synchronous AU transmission of $w_{a}s_{p}$ during the PU’s pilot training interval, the signal received at the BTS is then described as

(6) $$y_{b}=\sqrt{P_{p}}h_{pb}s_{p}+\sqrt{P_{a}}h_{ab}w_{a}s_{p}+n_{b}$$

where $P_{p}$ and $P_{a}$ are the transmit powers of the PU and the AU, respectively. The BTS cannot distinguish between the two signals; it perceives the sum of the paths as the legitimate PU’s channel. Unknowing of the AU’s signal manipulation, and to estimate the channel gain with the PU, the BTS correlates the received signal with the known pilot as

(7) $${\hat{h}}_{pb}=y_{b}\frac{{s_{p}}^{H}}{\sqrt{P_{b}}}=h_{pb}+\frac{\sqrt{P_{a}}}{\sqrt{P_{b}}}w_{a}h_{ab}+{\tilde{n}}_{b}$$

where ${\tilde{n}}_{b}$ is a noise vector component. Under high SNR and for similar values of $P_{b}$ and $P_{a}$, this estimate can be approximated as

(8) $${\hat{h}}_{pb}\approx h_{pb}+w_{a}h_{ab}.$$

The BTS uses the corrupted estimate ${\hat{h}}_{pb}$ to design the beamforming weight vector $w_{b}$. Using MRT, $w_{b}$ is found as $w_{b}=\frac{{\hat{h}}_{pb}^{H}}{∥{\hat{h}}_{pb}∥}$. Since $|w_{a}|=1$, the corrupted normalized BTS precoding weight becomes

(9) $$w_{b}=\frac{h_{pb}^{H}+w_{a}^{*}h_{ab}^{H}}{\sqrt{{∥h_{pb}∥}^{2}+{∥h_{ab}∥}^{2}+2ℜ\left\{w_{a}^{*}h_{ab}^{H}h_{pb}\right\}}}.$$

Thus, after the AU conducts the channel estimation poisoning, the BTS uses the incorrect channel measurements to unintentionally build the precoding weight that will lead to the AU conducting the PCA, as detailed in Section 4.

## 4. Pilot Contamination Attack

In the second stage of the attack, the AU utilizes how the BTS designs the precoding weight in (9) in order to alter the signal quality at the PU and/or at the AU. This leads to primary performance degradation and/or information leakage and the possibility of adversarial interception.

### 4.1. Attack Design

Consider the downlink transmission phase where the BTS intends to send signal *x* over the channel to the PU; hence, the BTS transmits *x*, along with precoding weight $w_{b}$ and transmit power $P_{b}$, with the intention to maximize the SNR at the PU. Given the transmitted signal of $\sqrt{P_{b}}w_{b}^{H}x$ and the channel reciprocality, the signal received by the legitimate PU becomes $y_{p}=\sqrt{P_{b}}w_{b}h_{pb}x+n_{p}$. Substituting $w_{b}$ in (9) leads to

(10) $$y_{p}=\sqrt{P_{b}}\frac{{∥h_{pb}∥}^{2}+w_{a}^{*}h_{ab}^{H}h_{pb}}{\sqrt{{∥h_{pb}∥}^{2}+{∥h_{ab}∥}^{2}+2ℜ\left\{w_{a}^{*}h_{ab}^{H}h_{pb}\right\}}}x+n_{p}.$$

To simplify the analysis, let

(11) $$\begin{array}{cc} w_{a} & =\alpha +j\beta \\ h_{ab}^{H}h_{pb} & =\gamma +j\delta \end{array}$$

where $\alpha ,\beta ,\gamma ,\delta \in R^{1\times 1}$. Given that $|w_{a}|=1$, α and β become subject to the $\alpha^{2}+\beta^{2}=1$ constraint. Thus, the normalized BTS precoding weight $w_{b}$ in (9) becomes

(12) $$w_{b}=\frac{h_{pb}^{H}+w_{a}^{*}h_{ab}^{H}}{\sqrt{{∥h_{pb}∥}^{2}+{∥h_{ab}∥}^{2}+2\left(\gamma \alpha +\delta \beta \right)}}.$$

The SNR at the PU, denoted as $\Gamma_{p}$, is degraded because the BTS unknowingly allocates a portion of its spatial power toward the attacker’s channel $h_{ab}$, effectively missteering the beam away from the PU’s optimal direction. Incorporating the expansion of $w_{b}$ into $y_{b}$ in (10), the actual SNR at the PU becomes

(13) $$\Gamma_{p}=\frac{P_{b}}{\sigma^{2}}\frac{\left({∥h_{pb}∥}^{4}+\gamma^{2}+\delta^{2}+2{∥h_{pb}∥}^{2}\left(\gamma \alpha +\delta \beta \right)\right)}{{∥h_{pb}∥}^{2}+{∥h_{ab}∥}^{2}+2\left(\gamma \alpha +\delta \beta \right)}.$$

Because the BTS aligns the phase of $w_{b}$ with the corrupted estimate (which contains $h_{ab}^{*}$), the AU receives a downlink signal as $y_{a}=\sqrt{P_{b}}w_{b}h_{ab}x+n_{a}$. Employing the value of $w_{b}$ from (9) leads to

(14) $$y_{a}=\sqrt{P_{b}}\frac{h_{pb}^{H}h_{ab}+{∥h_{ab}∥}^{2}w_{a}^{*}}{\sqrt{{∥h_{pb}∥}^{2}+{∥h_{ab}∥}^{2}+2ℜ\left\{w_{a}^{*}h_{ab}^{H}h_{pb}\right\}}}x+n_{a}.$$

Thus, using the definitions in (11), the normalized SNR at the AU, denoted as $\Gamma_{a}$, becomes

(15) $$\Gamma_{a}=\frac{P_{b}}{\sigma^{2}}\frac{\left({∥h_{ab}∥}^{4}+\gamma^{2}+\delta^{2}+2{∥h_{ab}∥}^{2}\left(\gamma \alpha +\delta \beta \right)\right)}{{∥h_{pb}∥}^{2}+{∥h_{ab}∥}^{2}+2\left(\gamma \alpha +\delta \beta \right)}.$$

### 4.2. Attack Optimization

Rewriting the expressions of $\Gamma_{p}$ in (13) and $\Gamma_{a}$ in (15) as

(16) $$\begin{array}{cc} \Gamma_{p} & =\frac{P_{b}}{\sigma^{2}({∥h_{pb}∥}^{2}+{∥h_{ab}∥}^{2}+2\left(\gamma \alpha +\delta \beta \right))}\left({∥h_{pb}∥}^{4}+\gamma^{2}+\delta^{2}+2{∥h_{pb}∥}^{2}\left(\gamma \alpha +\delta \beta \right)\right)\\ \Gamma_{a} & =\frac{P_{b}}{\sigma^{2}({∥h_{pb}∥}^{2}+{∥h_{ab}∥}^{2}+2\left(\gamma \alpha +\delta \beta \right))}\left({∥h_{ab}∥}^{4}+\gamma^{2}+\delta^{2}+2{∥h_{ab}∥}^{2}\left(\gamma \alpha +\delta \beta \right)\right) \end{array}$$

where $\alpha^{2}+\beta^{2}=1$, as indicated in (11). Following these SNR expressions, and to find the extrema of the function γα+δβ subject to the constraint $\alpha^{2}+\beta^{2}-1=0$, we define the Lagrangian function L as

(17) $$L\left(\alpha ,\beta ,\lambda \right)=\left(\gamma \alpha +\delta \beta \right)-\lambda \left(\alpha^{2}+\beta^{2}-1\right).$$

The first-order necessary conditions for an extremum are given by setting the partial derivatives to zero according to

(18) $$\begin{array}{cc} \frac{\partial L}{\partial \alpha } & =\gamma -2\lambda \alpha =0\Rightarrow \alpha =\frac{\gamma }{2\lambda }\\ \frac{\partial L}{\partial \beta } & =\delta -2\lambda \beta =0\Rightarrow \beta =\frac{\delta }{2\lambda }\\ \frac{\partial L}{\partial \lambda } & =\alpha^{2}+\beta^{2}-1=0. \end{array}$$

Substituting the expressions for α and β into the constraint equation leads to

(19) $${\left(\frac{\gamma }{2\lambda }\right)}^{2}+{\left(\frac{\delta }{2\lambda }\right)}^{2}=1\Rightarrow \frac{\gamma^{2}+\delta^{2}}{4\lambda^{2}}=1.$$

Solving for 2λ, we find

(20) $$2\lambda =\pm \sqrt{\gamma^{2}+\delta^{2}}.$$

Then, after employing the definitions of γ and δ in (11), the optimal coordinates $\left(\alpha^{*},\beta^{*}\right)$ are described using

(21) $$\begin{array}{cc} \alpha^{*} & =\pm \frac{\gamma }{\sqrt{\gamma^{2}+\delta^{2}}}\\ \; & =\pm \frac{ℜ\{h_{ab}^{H}h_{pb}\}}{|h_{ab}^{H}h_{pb}|}\\ \beta^{*} & =\pm \frac{\delta }{\sqrt{\gamma^{2}+\delta^{2}}}\\ \; & =\pm \frac{ℑ\{h_{ab}^{H}h_{pb}\}}{|h_{ab}^{H}h_{pb}|}. \end{array}$$

Note how this result satisfies the constraint of ${\alpha^{*}}^{2}+{\beta^{*}}^{2}=1$. Thus, if the objective of the AU is to maximize $\Gamma_{a}$, we choose the positive roots; however, if the objective is to minimize $\Gamma_{p}$, we choose the negative roots. Thus, when $\alpha =\frac{\gamma }{\sqrt{\gamma^{2}+\delta^{2}}}$ and $\beta =\frac{\delta }{\sqrt{\gamma^{2}+\delta^{2}}}$, we get

(22) $$w_{a_{\max}}=\frac{h_{ab}^{H}h_{pb}}{|h_{ab}^{H}h_{pb}|}$$

and the SNR values because of the PCA become

(23) $$\begin{array}{cc} \Gamma_{p_{\max}} & =\frac{P_{b}}{\sigma^{2}}\frac{{\left({∥h_{pb}∥}^{2}+\sqrt{\gamma^{2}+\delta^{2}}\right)}^{2}}{{∥h_{pb}∥}^{2}+{∥h_{ab}∥}^{2}+2\sqrt{\gamma^{2}+\delta^{2}}}\\ \Gamma_{a_{\max}} & =\frac{P_{b}}{\sigma^{2}}\frac{{\left({∥h_{ab}∥}^{2}+\sqrt{\gamma^{2}+\delta^{2}}\right)}^{2}}{{∥h_{pb}∥}^{2}+{∥h_{ab}∥}^{2}+2\sqrt{\gamma^{2}+\delta^{2}}}. \end{array}$$

On the other hand, the minimum values of the SNR can be achieved when $\alpha =-\frac{\gamma }{\sqrt{\gamma^{2}+\delta^{2}}}$ and $\beta =-\frac{\delta }{\sqrt{\gamma^{2}+\delta^{2}}}$, or when

(24) $$w_{a_{\min}}=-\frac{h_{ab}^{H}h_{pb}}{|h_{ab}^{H}h_{pb}|}.$$

In this case, the minimum SNR values are expressed as

(25) $$\begin{array}{cc} \Gamma_{p_{\min}} & =\frac{P_{b}}{\sigma^{2}}\frac{{\left({∥h_{pb}∥}^{2}-\sqrt{\gamma^{2}+\delta^{2}}\right)}^{2}}{{∥h_{pb}∥}^{2}+{∥h_{ab}∥}^{2}-2\sqrt{\gamma^{2}+\delta^{2}}}\\ \Gamma_{a_{\min}} & =\frac{P_{b}}{\sigma^{2}}\frac{{\left({∥h_{ab}∥}^{2}-\sqrt{\gamma^{2}+\delta^{2}}\right)}^{2}}{{∥h_{pb}∥}^{2}+{∥h_{ab}∥}^{2}-2\sqrt{\gamma^{2}+\delta^{2}}}. \end{array}$$

Note the tradeoff in attack design represented in (22)–(25), choosing to maximize $\Gamma_{a}$ also leads to higher values of $\Gamma_{p}$, and designing the PCA to minimize $\Gamma_{p}$ also reduces the potential value of $\Gamma_{a}$. The parametrized nature of the developed PCA, through choosing a specific value of $w_{a}$, allows the attacker to tune the attack to specifically target security metrics around information confidentiality or availability of the PU.

To formalize the information-theoretic implications of this threat configuration, we evaluate the achievable secrecy capacity ($R_{s}$) of the downlink channel. In physical-layer security paradigms, the secrecy capacity characterizes the maximum transmission speed at which data is reliably decoded by the legitimate PU while remaining perfectly secure from the eavesdropping AU [26,27]. Utilizing the normalized SNR tracking configurations derived in (13) and (15), the secrecy capacity is calculated using

(26) $$R_{s}=\max\left\{0,\;\log_{2}\left(1+\Gamma_{p}\right)-\log_{2}\left(1+\Gamma_{a}\right)\right\}.$$

This formulation highlights how the adversary uses its steering weight parameter $w_{a}$ to manipulate the spatial profiles of $\Gamma_{p}$ and $\Gamma_{a}$, compressing the reachable security bounds down to zero during aggressive attacks.

### 4.3. Algorithmic Attack Formulation

An algorithmic strategy for the parametrized PCA is developed in Algorithm 1. In this strategy, the AU collects the CSI; estimates the pilot signals, transmit powers, and channel vectors; and calculates the attack weight vector $w_{a}$ and its transmit power. During the channel estimation poisoning stage, the AU synchronizes its transmission with the PU and injects $\sqrt{P_{a}}w_{a}s_{p}$ into the channel to sway the pilot estimates made by the BTS. As the BTS moves to the downlink transmission and precoding phase, the AU receives the missteered transmission and conducts the security attack. The cycle repeats until the AU concludes the PCA.
**Algorithm 1** Pilot Contamination Attack Strategy**while** TRUE **do** Measure: $y_{p}$, $y_{a}$. Estimate: $s_{p}$, $s_{b}$, $P_{p}$, $P_{b}$. Estimate: $h_{ab}$, $h_{pb}$. Calculate: $w_{a}$, $P_{a}$. **if** Estimation Poisoning Stage **then**  Synchronize: Transmission Frame.  Transmit: $\sqrt{P_{a}}w_{a}s_{p}$. **end if** **if** Contamination Attack Stage **then**  Receive: $y_{a}$.  Calculate: $\Gamma_{a}$.  Conduct: Security Attack. **end if** **if** PCA is over **then**  Break. **end if****end while**

To evaluate the operational feasibility of the threat agent, we characterize the computational complexity of Algorithm 1 using Big-O notation as a function of the number of base transceiver antennas *M*. The measurement and scalar frame synchronization steps require basic sampling tracking, scaling as O(1). The core computational bottleneck resides within the vector estimation and optimization steps. Isolating and estimating the multi-antenna channel parameters $h_{ab}$ and $h_{pb}$ requires vector correlation and division loops matching the size of the antenna array, yielding a complexity of O(M). Subsequently, evaluating the optimal complex weight allocation $w_{a}$ in (22) or (24) involves a vector inner-product multiplication ($h_{ab}^{H}h_{pb}$) and scalar normalization, which adds an operational cost of O(M). Because these steps occur sequentially within each tracking iteration loop, the total computational complexity per frame coherence interval scales linearly as O(M). This linear dependence demonstrates that the parameterized threat model imposes minimal processing overhead on the adversary, enabling real-time execution even within modern large-scale multi-antenna systems.

### 4.4. Comparison with the Baseline Case

Consider a baseline case where there is no PCA (i.e., $w_{a}=0$), and assume a perfect CSI at the BTS (i.e., ${\hat{h}}_{pb}=h_{pb}$). This leads to the BTS’s precoding gain as $w_{b}=\frac{h_{pb}^{H}}{∥h_{pb}∥}$. In this scenario, the received signal at the PU is $y_{p}=\sqrt{P_{b}}∥h_{pb}∥x+n_{p}$. Thus, the primary SNR becomes

(27) $$\Gamma_{p}=\frac{P_{b}}{\sigma^{2}}{∥h_{pb}∥}^{2}.$$

Note that ${∥h_{pb}∥}^{2}$ follows a Chi-squared distribution with a mean of *M*, where *M* is the number of BTS antennas. On the other hand, the received signal at the adversary becomes $y_{a}=\sqrt{P_{b}}\frac{h_{pb}^{H}h_{ab}}{∥h_{pb}∥}x+n_{a}$. This leads to an SNR value at the AU as

(28) $$\Gamma_{a}=\frac{P_{b}}{\sigma^{2}}\frac{{\left|h_{pb}^{H}h_{ab}\right|}^{2}}{{∥h_{pb}∥}^{2}}.$$

A channel outage occurs for the PU when its instantaneous channel capacity falls below a threshold required to support its transmission rate ($R_{p}$) [28]. In other words, a primary channel outage occurs when $\log_{2}\left(1+\Gamma_{p}\right)<R_{p}$ [29] or when $\Gamma_{p}<2^{R_{p}}-1$. Given the PU’s SNR value in (27), a channel outage occurs in the baseline case for the PU when

(29) $${∥h_{pb}∥}^{2}<\frac{\sigma^{2}}{P_{b}}\left(2^{R_{p}}-1\right).$$

### 4.5. Sensitivity to CSI Imperfections

To rigorously evaluate the feasibility of the PCA, it is necessary to distinguish the inherent channel estimation errors at the BTS from the localized CSI imperfections experienced by the AU. Furthermore, we must isolate these baseline conditions from the resulting composite error caused by the attack. Define the AU’s localized CSI imperfection using a Gauss–Markov error model, where in practice, the AU does not possess perfect knowledge of the adversarial link to the BTS; instead, the AU operates using a degraded channel estimate as

(30) $${\hat{h}}_{ab}=\sqrt{1-\epsilon^{2}}h_{ab}+\epsilon e_{a}$$

where ϵ∈[0,1] parameterizes the severity of the CSI degradation, and $e_{a}$ is an independent complex Gaussian error vector local to the AU. Consequently, the AU must compute the optimal attack weight vector $w_{a}$ using this corrupted estimate of the channel, yielding a sub-optimal weight ${\tilde{w}}_{a}$.

Let $e_{b}$ denote the benign, ambient estimation noise present at the BTS when attempting to estimate the PU channel under normal operating conditions, prior to any adversarial interference. Thus, the final estimation error observed at the BTS during the attack is a composite of the deterministic, structural error injected during the channel estimation poisoning stage through ${\tilde{w}}_{a}$ and the ambient estimation noise $e_{b}$. The sensitivity of the attack’s efficacy to the parameter ϵ is numerically validated in Section 5, demonstrating the impact of imperfect CSI on the cybersecurity outcomes in the pilot contamination phase.

## 5. Numerical Results

In this section, we demonstrate the efficacy of the PCA through representative numerical examples. Specifically, we consider the impact of the PCA on the SNR, bit error rate, and outage probability values for the users in the system during the downlink transmission interval. The numerical results show these metrics for three cases of attack as

(31) $$w_{a}=\left\{\begin{array}{cc} w_{a_{\max}} & \mathrm{Maximum}\;\mathrm{SNR}\;\mathrm{Attack}\;\mathrm{Profile}\\ w_{a_{\min}} & \mathrm{Minimum}\;\mathrm{SNR}\;\mathrm{Attack}\;\mathrm{Profile}\\ 0 & \mathrm{No}\;\mathrm{PCA}\;\mathrm{Attack}. \end{array}\right.$$

$w_{a}=0$ signifies the case when there is no attack, and it will be used as a benchmark to compare the impact of the PCA on the PU and the AU.

Recall the elements of the CIA triad: confidentiality, integrity, and availability. In the context of this article, the SNR value of PU’s intended signal measured at the AU acts as a measure of information confidentiality since it indicates the possibility of the AU being able to intercept and eavesdrop on PU’s communication. On the other hand, the bit error rate of the PU’s downlink signal is used as a metric of information integrity since it measures the signal corruption due to the PCA. Finally, the outage probability of the PU’s downlink channel acts as a measure of information availability since it quantifies the percentage of time the PU is not able to decode its received signal.

For the numerical results in this section, Monte Carlo simulations are used to plot the PCA’s efficacy metrics versus the base SNR (i.e., $\frac{P_{b}}{\sigma^{2}}$). In the simulation environment, the BTS has M=4 antennas, the pilot signal $s_{p}$ has four bits, a binary phase shift keying (BPSK) modulation is used to transmit the data over the channel, and the downlink transmission rate is $R_{p}=1$ bit/sec/Hz. The communication environment is simulated using a Rayleigh block-fading model.

**Figure 3:**
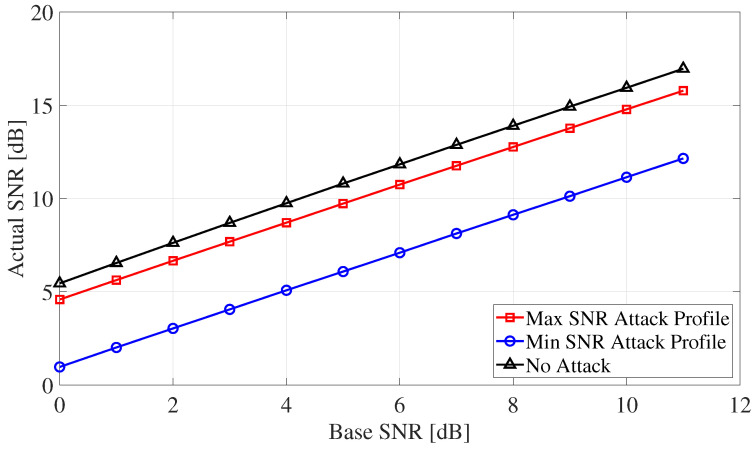
Primary user’s actual SNR.

Figure 3 illustrates the actual SNR attained by the PU during the PCA. Note that when there is no attack, the actual SNR scales linearly with the base SNR with a beamforming gain of $10\log_{10}M$. However, the PCA results in reduced SNR values for the PU; for the case when the AU uses $w_{a_{\min}}$ when orchestrating the attack, the PU loses around 5 dBs of actual SNR. On the other hand, when $w_{a_{\max}}$ is used, the loss in dBs is still noticeable yet less drastic.

**Figure 4:**
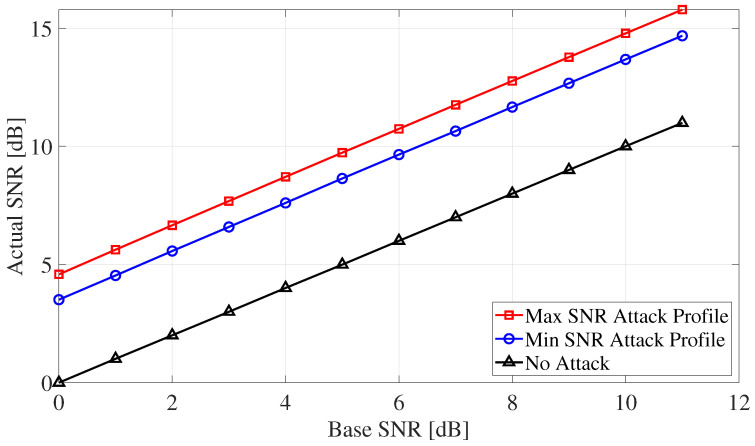
Adversary user’s actual SNR.

The actual SNR values for the AU during the attack are shown in Figure 4. As observed, compared to the case of no PCA, the AU can achieve around 5 dBs of SNR gain when $w_{a_{\max}}$ is used. Less SNR gain is achieved, yet still substantial, when $w_{a_{\min}}$ is used to construct the attack. The results of this figure emphasize the usefulness of the PCA in enabling the AU to receive a quality reception that was initially designed for the PU. For example, comparing Figure 3 and Figure 4 at a base SNR of 10 dBs, both the PU and the AU have similar actual SNR around 15 dBs. This result can be interpreted as a violation of the information confidentiality of the PU.

**Figure 5:**
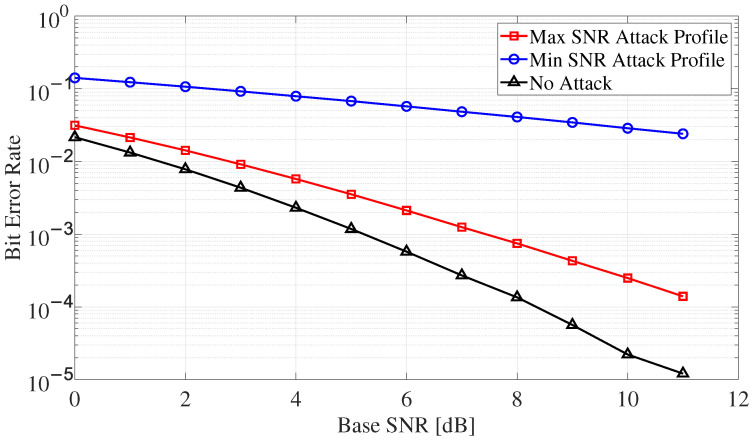
Primary user’s bit error rate.

Figure 5 displays the average bit error rate of the PU versus the base SNR. The no-attack case demonstrates the bit error rate of BPSK modulation in a Rayleigh block-fading environment. However, when the AU conducts the PCA on the PU’s downlink communication, the bit error rate substantially increases, indicating an unfavorable communication environment for the PU. This is especially clear when the AU chooses $w_{a_{\min}}$ during the channel estimation poisoning stage. The deterioration of the bit error rate can be understood as an infraction on the information integrity of the PU’s downlink communication activity.

**Figure 6:**
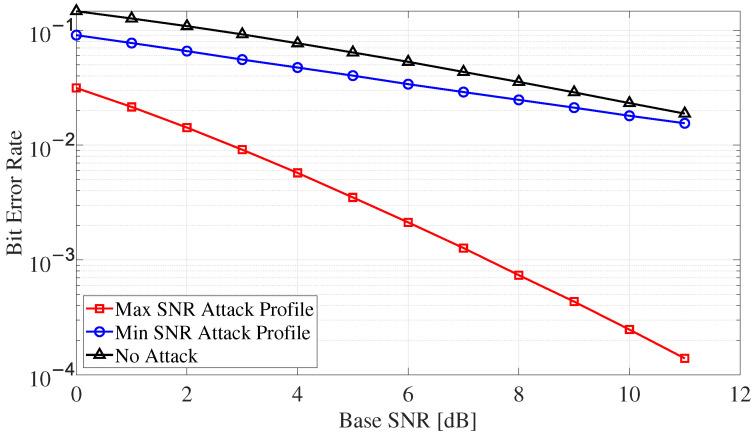
Adversary user’s bit error rate.

The bit error rate calculated at the AU’s side is shown in Figure 6. This result indicates the ability of the AU to decode the downlink messages directed at the PU. Consistent with the findings of Figure 4, the AU’s best performance metrics (i.e., lower bit error rate values) are achieved when $w_{a_{\max}}$ is used during the channel estimation poisoning stage of the attack.

**Figure 7:**
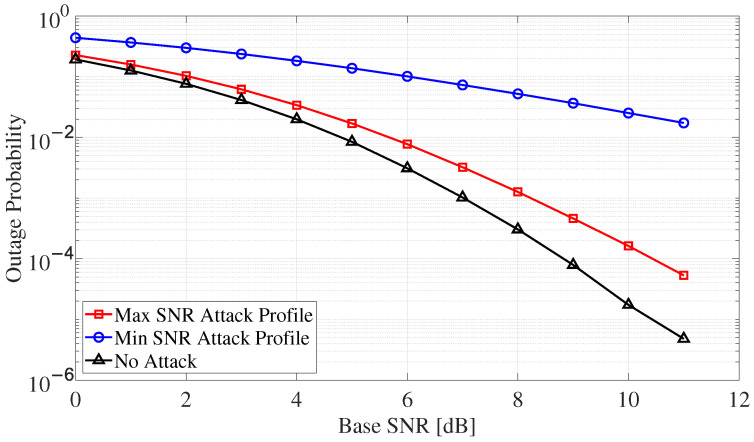
Primary user’s outage probability.

A channel outage can be used as a measure of data availability for the PU, where more outage indicates less ability to access the communicated information when needed. Figure 7 displays the channel outage probability of the PU’s downlink. While the outage probability improves with increasing the base SNR values, it is noted that this improvement is hindered by the PCA. Specifically, when the AU employs $w_{a_{\min}}$ during the channel estimation stage, the PU sustains more pronounced channel outages compared to the case when there is no PCA.

**Figure 8:**
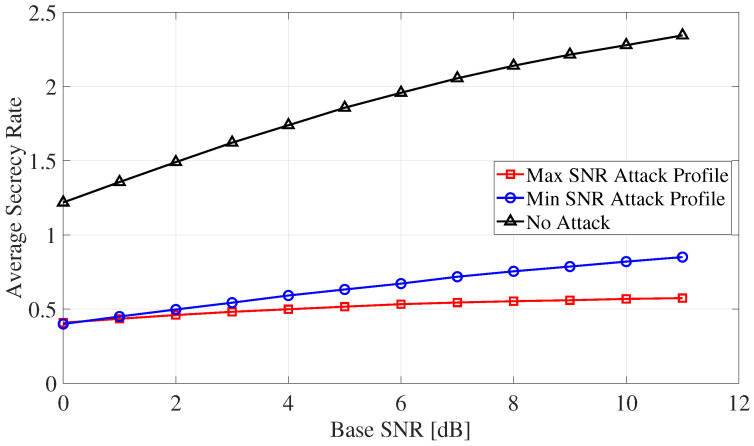
Primary user’s secrecy rate.

Figure 8 illustrates the system’s average secrecy rate ($R_{s}$) as a function of the base SNR. When the system operates free of adversarial activity (i.e., $w_{a}=0$), the secrecy rate scales logarithmically with base SNR, as documented in (26) and inferred from (27) and (28), capitalizing on uncontaminated beamforming directionality. However, when the active adversary orchestrates a PCA using $w_{a_{\max}}$, the available secrecy capacity drops dramatically, approaching a lower floor as the base SNR scales up. This explicit collapse underscores that maximizing the adversary’s received SNR successfully forces critical primary information leakage, confirming the performance trade-offs established in our parametric threat model.

**Figure 9:**
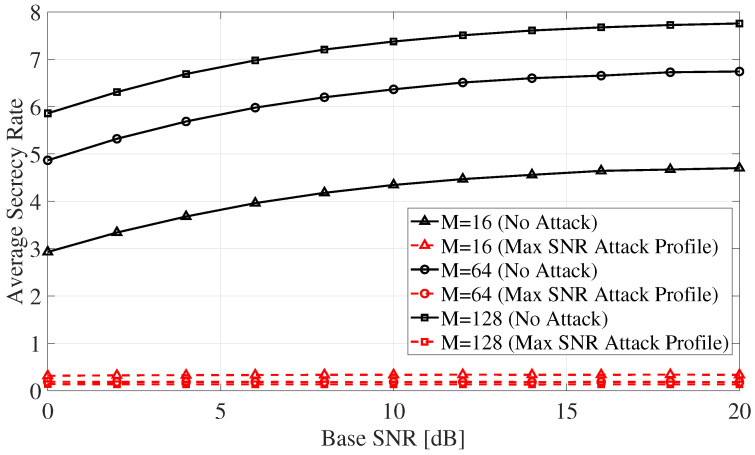
Performance evaluation under MIMO configuration.

To further validate the scalability of the proposed attack strategy and assess its performance bounds under realistic operational constraints, Figure 9 illustrates the average secrecy rate as a function of the base SNR across expanded BTS antenna configurations (M=16,64, and 128). In the absence of an adversary, scaling the antenna array dimension yields significant performance gains, driving up the achievable secrecy capacity via highly directive beamforming. However, when the active adversary launches the optimized PCA under the Max SNR attack profile, the system’s secrecy rate experiences a severe collapse, dropping to a low floor across all evaluated antenna numbers. This phenomenon reveals that MIMO configurations do not inherently provide immunity to highly synchronized physical layer injections; instead, the scaling benefits are neutralized by the malicious PCA orchestrated by the AU.

**Figure 10:**
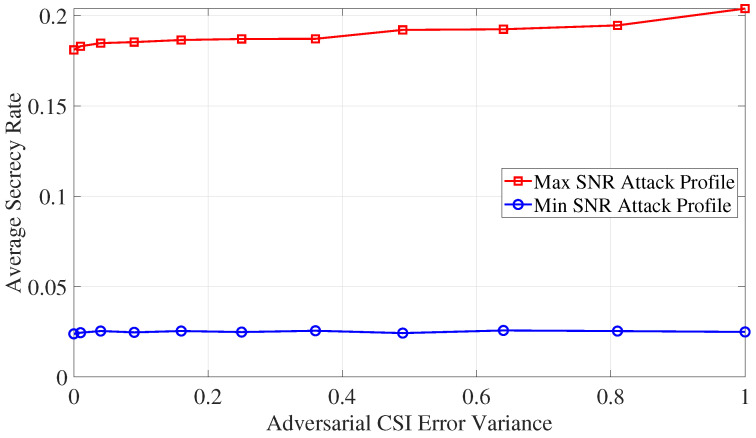
Performance evaluation under imperfect adversarial channel knowledge.

Furthermore, Figure 10 presents a sensitivity analysis addressing the practical limitations of the adversary’s capabilities by introducing a Gauss–Markov error framework to model imperfect adversarial CSI. The average secrecy rate is tracked against the adversarial channel estimation error variance ($\epsilon^{2}$) at a base SNR of 10 dB and M=64. Under the Max SNR attack profile, a distinct degradation trend in the attack’s overall efficacy is observed as $\epsilon^{2}$ scales from 0 (perfect knowledge) to 1 (complete statistical uncertainty). Specifically, as the adversary’s channel estimates deteriorate, its weight optimization misaligns with the legitimate subspace channels, causing a gradual upward recovery of the PU’s secrecy rate. However, the Min SNR attack profile remains largely bounded at a minimal secrecy floor due to its distinct optimization constraints. This evaluation confirms that while the proposed threat vector introduces severe systemic vulnerabilities, its real-world implementation exhibits a quantifiable sensitivity to the accuracy of the AU’s channel estimation process.

## 6. Discussion

Pilot contamination transforms the BTS from a secure transmitter into an unintentional accomplice of the adversary, creating a high-gain channel for eavesdropping while starving the legitimate link of the intended power. The PCA can lead to infringements on the security metrics of the PU through increased SNR values for the AU and through reduced SNR and increased bit error rate and channel outages for the PU.

### 6.1. Attack Impact

Recall that the AU’s PCA strategy is to create a misalignment in the BTS precoding process to direct transmitted downlink energy away from the intended PU and toward the adversary. By capturing this diverted signal, the AU aims to breach the PU’s information confidentiality while simultaneously degrading the PU’s signal quality to infringe upon its information integrity and availability.

In the context of this work, the impact on the PU’s information confidentiality can be assessed through the increased SNR at the AU, as a stronger signal enhances the adversary’s ability to decode the intercepted downlink transmission. On the other hand, the impact on information integrity can be quantified through the reduced SNR at the legitimate PU’s downlink signal, which leads to a higher bit error rate and a higher rate of dropped packets. Further, the impact on information availability can be evaluated through the reduced SNR at the PU, which leads to higher channel outages and the inability of the PU to correctly decode the downlink signals.

Finally, it is observed that when the AU uses $w_{a_{\max}}$ to poison the channel estimation at the BTS, better signal metrics are found at the PU and AU; on the other hand, using $w_{a_{\min}}$ during the channel estimation stage leads to more deterioration in the PU’s signal. In all cases, the PU’s signal quality and consequently its security metrics are worse off because of the PCA compared to the case of no attack.

### 6.2. Directions for Future Defense Strategies

Securing modern MIMO infrastructures requires a holistic physical-layer security framework that integrates detection vectors and active mitigation paradigms [19]. In a single-cell block-fading environment lacking spatial line-of-sight components, separating the PU’s channel from the AU’s poisoned observation relies heavily on exploiting statistical, temporal, and algorithmic asymmetries.

To detect channel poisoning within short coherence intervals, the BTS can identify statistical anomalies by analyzing inconsistencies between the instantaneous received signal covariance and long-term CSI trends. Furthermore, leveraging random training sequences establishes an asymptotic bound for clean observations, effectively exposing synchronized spoofing attempts [30]. In non-orthogonal multiple access networks, overlapping resource blocks intensify PCA vulnerabilities. This can be countered by implementing generalized likelihood ratio tests and energy-detection hypothesis frameworks [31,32]. Evaluating received pilot energy vectors against established bounds, while integrating statistical-based energy detectors, maintains high detection rates despite severe channel fluctuations [33].

To counter adaptive, intelligent adversaries, threshold defenses are increasingly augmented with generative adversarial networks (GANs) and game theory [34,35]. By training a GAN discriminator exclusively on uncompromised pilot observations, the BTS learns the baseline distribution of the PU’s channel to flag real-time adversarial micro-deviations [36]. Further, game-theoretic formulations can model the attack–defense cycle as a dynamic zero-sum game, allowing the network to adaptively adjust detection thresholds and power allocation policies to match the evolving strategy of the attacker.

Following attack detection, active mitigation strategies should preserve downlink confidentiality and integrity. To disrupt the synchronization required for a successful two-stage attack, wireless systems should replace predictable static pilots with dynamic pilot hopping and user-specific sequence permutations [37]. Synchronizing a pseudorandom pilot-hopping seed via higher-layer encryption allows the PU and BTS to continuously alter pilot patterns, preventing the AU from aligning its transmission within the target coherence interval.

When the contaminated CSI cannot be entirely discarded, the precoding stage can be algorithmically shielded. Robust precoding algorithms project downlink transmit signals onto the orthogonal complement of the suspected adversarial subspace, executing directional nulling to protect the PU’s confidentiality. Furthermore, integrating deep-learning- or GAN-based classification directly into the precoding algorithm enables defensive gating; if a pilot sequence exhibits a high probability of contamination, the BTS can dynamically suppress precoding weights or trigger an immediate pilot reallocation cycle. Beyond training phase modifications, integrating artificial noise injection and covert communications ensures information-theoretic security even if beamforming weights are biased. Embedding a controlled level of degradation into the null-space of the PU’s channel can diminish the AU’s SNR posture while preserving link availability for the intended PU [38].

Transitioning these defensive strategies to production-grade physical layers presents a few primary research challenges. First, under deep Rayleigh fading, high mobility, or low SNR, distinguishing malicious contamination from severe multipath interference is difficult given tight channel coherence constraints. Second, advanced machine learning detectors and iterative precoding optimization algorithms impose heavy computational burdens, requiring careful trade-offs with latency constraints. Third, large-scale single-cell coordination is needed to handle inter-cell pilot reuse and roaming attackers without incurring excessive overhead. Finally, bridging physical-layer detection vectors with higher-layer cryptographic protocols necessitates standardized cross-layer interfaces and unified signaling formats to trigger real-time security remediation.

While this work establishes the foundational vulnerability in a baseline single-cell environment, extending this mathematical framework represents a critical direction for future work. Specifically, future research should evaluate the proposed attack vector under expanded network architectures, incorporating multi-cell pilot reuse patterns, user mobility profiles, and large-scale path loss. Furthermore, the mitigation strategies discussed above require rigorous analytical and numerical validation within these dynamic, multi-cell threat models to serve as concrete defensive solutions.

## 7. Conclusions

In this article, we deconstruct the mechanics and security implications of PCAs in MIMO systems through a parameterized, two-stage operational threat model. By breaking the attack down into an initial channel estimation poisoning stage followed by an adaptive contamination stage, we provide a systematic framework to quantify adversarial exploitation. Our analysis evaluates this physical-layer threat through the lens of the CIA triad, mapping the direct mathematical relationship between the adversary’s security gains and the degradation of the PU’s signal quality. This parametric formulation demonstrates that active poisoning during the uplink training phase in a single-cell block-fading environment can compromise downlink information confidentiality and availability without requiring prior channel knowledge or high-complexity hardware from the AU.

Building upon this threat model, we discuss a defensive roadmap that transitions from passive detection to active physical-layer mitigation, and we discuss several mechanisms to shield the downlink transmission and maintain information-theoretic security even under compromised CSI estimations. Ultimately, while these defensive strategies offer robust theoretical protection, transitioning them to production-grade wireless architectures exposes critical open research horizons. Future work should focus on resolving the strict trade-offs between computational complexity and latency constraints, enhancing detection robustness under deep fading and high-mobility scenarios, and standardizing cross-layer signaling interfaces. Resolving these challenges is essential to establish resilient, self-defending physical layers capable of securing next-generation wireless communication networks against sophisticated physical-layer threats.

## Data Availability

The original contributions presented in this study are included in the article. Further inquiries can be directed to the corresponding author.
